# Ahmed Hankir: fighting mental health stigma

**DOI:** 10.2471/BLT.22.030822

**Published:** 2022-08-01

## Abstract

Ahmed Hankir talks to Vijay Shankar Balakrishnan about his work at the frontier between the arts and clinical psychiatry to combat stigma.

*Q: What inspired you to become a psychiatrist?*

A: I had always wanted to be a doctor but had originally intended to be a heart surgeon. My interest in psychiatry was born out of my own lived experience of a mental health condition.

*Q: Can you talk about that?*

A: My parents fled to Northern Ireland to escape the civil war in Lebanon, and I was born in Belfast in 1982. My mother always says it was like “jumping out of the frying pan and into the fire” because they arrived in Belfast during the brutal conflict known as The Troubles. We then moved to England, eventually returning to war-torn Lebanon in 1995. Because educational opportunities were limited in Lebanon at that time, I was compelled to return to the UK with my twin brother in 2000 to finish my studies. My dream was to get into medical school, but I discovered that my Lebanese qualifications were not recognized in the UK, so I had to do A levels at a “Sixth Form” College, working full-time in a supermarket to pay my way. This was one of the toughest times in my life. And it wasn’t helped by the prejudice I had to deal with. I remember the Head of the Sixth Form asking me what I wanted to do with my life and when I said I wanted to become a doctor she laughed in my face. She said I’d never get into medical school and that I should choose another course. She made me feel like I was this dirty little immigrant with delusions of grandeur. I can’t deny it, her words hurt me a lot. But I dug deep and went on to receive straight A grades and matriculated into Manchester Medical School.

*Q: Did things get easier at medical school?*

A: (laughing) Not really! I was having to study full-time and support myself doing different jobs, including flipping burgers, scrubbing floors and wiping tables to pay for food and a roof over my head. It was hard. Not just from the point of view of the long hours I had to work but also the dehumanizing contempt and racism that I had to deal with on a daily basis. When I was cleaning floors, I couldn’t understand why people didn’t acknowledge me when I said, “good morning”. Despite my best efforts to integrate, I always felt excluded, like I never belonged. I experienced an identity crisis associated with emotional turmoil and psychological distress during medical school. Then, three years into my degree I woke up to the news that Beirut had been bombed. That was a turning point for me. I could not believe the horrible images emerging from what I had come to consider my hometown in Lebanon. It really affected me, and I plunged into a period of severe melancholia. I didn’t even want to get out of bed, let alone go to classes. I also experienced somatic symptoms including excruciating chest pain. I felt like I couldn’t breathe. On top of everything else, instead of being supported by my peers, I was shunned, even ridiculed. I was told to take a year out and then I lost my accommodation. It was a horrible time. In my isolation and despair, I contemplated ending my own life. My Islamic faith deterred me from following through, thank God.

“Wounded Healers are [..] tremendously effective and empathic.”

*Q: How did you begin the process of recovery?*

A: In the end, I went to the imam at my local mosque, who urged me to get help from an NHS (National Health Service) psychiatrist. So that’s what I did. We co-produced a care plan and slowly but surely, I started to recover. And during my recovery something interesting happened. In the depths of my depression, I had started taking a real interest in films and books. I was watching a variety of movies, reading novels and poetry. I identified with the protagonists especially when they were the victims of injustice and discrimination and found that identification was therapeutic and cathartic. When the adverse effects of the psychotropic drugs I was taking became intolerable, I started trying to help myself by reciting poetry, and passages from books, and lines from films – declaiming them, performing them. I felt empowered and dignified. They lifted me up. I also modified my lifestyle, eating healthily and really exercising regularly, including running up to 20 kilometres a day.

*Q: How did your experience influence your thinking and your work?*

A: Well, first it gave me the confidence and the desire to finish my studies. But my encounter with art changed my thinking too. I completed an out-of-area Special Student Component in Cambridge University under the supervision of Dr Rashid Zaman as part of my studies on the association between the artistic temperament and profound oscillations in mood. That really piqued my interest in art therapy, and it continues to be one of my core interests. My experience of stigma also fed into my work. After I got my medical degree in 2011, I continued with two years of placements in the NHS, while at the same time developing my own approach to combating the stigma associated with mental health conditions, particularly in the medical profession. I published papers, wrote book chapters, co-edited textbooks, and started giving talks while developing my Wounded Healer anti-stigma presentation.

*Q: What do you mean by “Wounded Healer”?*

A: The basis of the idea – which is often credited to the great psychoanalyst Carl Jung – is that the healer is compelled to heal because of his/her own wounds. A corollary idea is that, in order to heal, the healer must come face to face with his/her own wounds. There has been a tendency in the medical profession – and I am speaking broadly here – to represent the healer or doctor as somehow infallible or invincible, and invulnerable, and to shame those doctors who show fallibility or vulnerability. It is one of the reasons doctors suffer burnout before they seek help. One of the important messages of my presentation is that Wounded Healers are not only worthy of respect but are in fact tremendously effective and empathic healers because of the insight and compassion they bring to their interactions with patients. The Wounded Healer presentation derives from a psycho-educational approach to mental health that embraces the non-pharmacological model of psychiatry. It is at its core a theatrical intervention that blends the power of performing arts and storytelling with psychiatry. I first entertain and engage audiences by re-enacting scenes from famous films and reciting poetry and then once engaged, I educate them with the facts about stigma, while sharing aspects of my personal journey as an impoverished, suicidal, male medical student, a man from a minority ethnic background, who went from being isolated and suicidal to becoming an empowered mental health practitioner and advocate.

“Stigma research is itself stigmatized.”

*Q: To what degree is racial discrimination a factor in the mental health of stigmatized populations?*

A: The available evidence suggests that it is a key determinant. I suspect that racism is one of the main reasons people of colour are overrepresented as patients and inmates in mental health care services and the criminal justice system. The stigmatization of people for their religious beliefs is another determinant. This is a growing problem not just in the UK, but in other high-income countries too. Professor Jonas Kunst and colleagues from the University of Oslo, Norway, have conducted important research in this area, for example developing and validating a Perceived Islamophobia Scale which indicated an association between subjects’ perceived stigmatization and their psychological distress. Needless to say, such psychosocial stressors are generally accompanied by others such as poverty, homelessness, loneliness, and unemployment, all of which can increase the incidence of mental health conditions and suicidal behaviours. The fact that people subject to these stressors are less likely to be able to access mental health services only exacerbates the problem.

*Q: How should psychosocial approaches be integrated into the care provided by institutions?*

A: To begin with we need quantitative and qualitative data to show the extent to which they work. The literature regarding the role non-pharmacological interventions can play in improving outcomes in people with mental health conditions is growing and includes papers I have published that are based on assessing the impact of The Wounded Healer, but more research is needed to fully understand what works and to establish best practice. Unfortunately, stigma research is itself stigmatized in the psychiatric community. If you write a grant application for research on a new psychotropic drug, you stand a good chance of receiving funding, but someone trying to identify innovative ways to reduce mental health-related stigma will struggle. Needless to say, there are market forces at work here which is why making human rights-based arguments in support of stigma-related research is so important. In this regard, I would like to mention WHO’s QualityRights initiative which has been developed by the Department of Mental Health and Substance Use. Eliminating stigma and discrimination and promoting human rights are among the aims of the initiative. I have been collaborating closely with WHO’s Dr Michelle Funk on the project and am hopeful that it will be adopted worldwide.

We also need to do more to teach medical students about how devastating mental health-related stigma can be and what they can do to challenge and reduce it. As part of my anti-stigma work, I also advocate for the inclusion of stigma studies in medical school and am happy to report that four universities in the UK have now adopted the Wounded Healer presentation into their curricula.

The potential benefits of new approaches are enormous. To give just one example, in Zimbabwe Professor Dixon Chibanda developed an approach to mental health care that involves training grandmothers to deliver low-intensity psychological therapy. A randomized clinical trial published in the December 2016 issue of the *Journal of the American Medical Association* revealed that grandmothers were better than doctors at treating depression! The result didn’t surprise me. The compassion, non-judgemental insight and understanding of those grandmothers was clearly very powerful. I can imagine similar approaches being used in conflict zones delivered by non-specialists grounded on humanity, human rights and compassion. That, in my humble opinion, is one of the ways we can help reduce the pervasive treatment gap in mental health.

